# Neuroprotective Effects of Tryptanthrin-6-Oxime in a Rat Model of Transient Focal Cerebral Ischemia

**DOI:** 10.3390/ph16081057

**Published:** 2023-07-25

**Authors:** Mark B. Plotnikov, Galina A. Chernysheva, Vera I. Smol’yakova, Oleg I. Aliev, Anna M. Anishchenko, Olga A. Ulyakhina, Eugene S. Trofimova, Anastasia A. Ligacheva, Nina D. Anfinogenova, Anton N. Osipenko, Anastasia R. Kovrizhina, Andrei I. Khlebnikov, Igor A. Schepetkin, Anastasia G. Drozd, Evgenii V. Plotnikov, Dmitriy N. Atochin, Mark T. Quinn

**Affiliations:** 1 Department of Pharmacology, Goldberg Research Institute of Pharmacology and Regenerative Medicine, Tomsk National Research Medical Center, Russian Academy of Sciences, Tomsk 634028, Russia; mbp2001@mail.ru (M.B.P.); bona2711@mail.ru (G.A.C.); light061265@mail.ru (V.I.S.); oal67@yandex.ru (O.I.A.); nuska-80@mail.ru (A.M.A.); olya.andreevna2000@mail.ru (O.A.U.); eugenie76@mail.ru (E.S.T.); vittelli@mail.ru (A.A.L.); 2 Faculty of Radiophysics, National Research Tomsk State University, Tomsk 634050, Russia; 3 Department of Pharmacology, Siberian State Medical University, Tomsk 634050, Russia; osipenko-an@mail.ru; 4 Cardiology Research Institute, Tomsk National Research Medical Center, Russian Academy of Sciences, Tomsk 634012, Russia; cardio.intl@gmail.com; 5 Kizhner Research Center, Tomsk Polytechnic University, Tomsk 634050, Russia; anaskowry@gmail.com (A.R.K.); aikhl@chem.org.ru (A.I.K.); 6 Department of Microbiology and Cell Biology, Montana State University, Bozeman, MT 59717, USA; igor@montana.edu; 7 Research School of Chemistry and Applied Biomedical Sciences, Tomsk Polytechnic University, Tomsk 634050, Russia; agd7@tpu.ru (A.G.D.); plotnikov.e@mail.ru (E.V.P.); 8 Mental Health Research Institute, Tomsk National Research Medical Center, Russian Academy of Sciences, Tomsk 634014, Russia; 9 Cardiovascular Research Center, Cardiology Division, Massachusetts General Hospital, Harvard Medical School, Charlestown, MA 02115, USA

**Keywords:** anti-inflammatory activity, antioxidant activity, *c*-Jun N-terminal kinase, focal cerebral ischemia, ischemic stroke, neuroprotective activity, tryptanthrin-6-oxime

## Abstract

The activation of *c*-Jun N-terminal kinase (JNK) plays an important role in stroke outcomes. Tryptanthrin-6-oxime (TRYP-Ox) is reported to have high affinity for JNK and anti-inflammatory activity and may be of interest as a promising neuroprotective agent. The aim of this study was to investigate the neuroprotective effects of TRYP-Ox in a rat model of transient focal cerebral ischemia (FCI), which involved intraluminal occlusion of the left middle cerebral artery (MCA) for 1 h. Animals in the experimental group were administered intraperitoneal injections of TRYP-Ox 30 min before reperfusion and 23 and 47 h after FCI. Neurological status was assessed 4, 24, and 48 h following FCI onset. Treatment with 5 and 10 mg/kg of TRYP-Ox decreased mean scores of neurological deficits by 35–49 and 46–67% at 24 and 48 h, respectively. At these doses, TRYP-Ox decreased the infarction size by 28–31% at 48 h after FCI. TRYP-Ox (10 mg/kg) reduced the content of interleukin (IL) 1β and tumor necrosis factor (TNF) in the ischemic core area of the MCA region by 33% and 38%, respectively, and attenuated cerebral edema by 11% in the left hemisphere, which was affected by infarction, and by 6% in the right, contralateral hemisphere 24 h after FCI. TRYP-Ox reduced *c*-Jun phosphorylation in the MCA pool at 1 h after reperfusion. TRYP-Ox was predicted to have high blood–brain barrier permeability using various calculated descriptors and binary classification trees. Indeed, reactive oxidant production was significantly lower in the brain homogenates from rats treated with TRYP-Ox versus that in control animals. Our data suggest that the neuroprotective activity of TRYP-Ox may be due to the ability of this compound to inhibit JNK and exhibit anti-inflammatory and antioxidant activity. Thus, TRYP-Ox may be considered a promising neuroprotective agent that potentially could be used for the development of new treatment strategies in cerebral ischemia.

## 1. Introduction

Stroke is the second leading cause of death and a major cause of disability worldwide [1]. Strokes can be classified as ischemic and hemorrhagic, with most strokes (87%) being ischemic strokes [2]. Despite significant progress in our understanding of ischemic stroke mechanisms [3,4,5], current clinical trials evaluating neuroprotective agents in patients with ischemic stroke have not had much success, and none of these neuroprotectants have moved into clinical practice [6]. Thus, the development of new and more effective approaches for the prevention and treatment of stroke is essential [7].

Two main therapeutic strategies are used during the acute phase of stroke [8]. The first strategy is recanalization of the occluded vessel and reperfusion. However, despite recanalization, many patients do not achieve good functional recovery. Moreover, the narrow time window of thrombolytic therapy (<4.5 h) limits the clinical use of this modality [9]. The second therapeutic strategy for ischemic stroke involves the administration of neuroprotective therapy to prevent metabolic changes involved in the ischemic cascade [9,10]. The failure of neuroprotectants in translational studies led to the substantial revision of animal models, targeted study designs, and the implementation of innovative research technologies for stroke [11]. The complex nature of cerebral ischemia–recirculation pathogenesis [5] provides the rationale behind the search for candidate neuroprotectants among compounds with multitarget activity [12,13]. Although clinical trials have identified a few drugs with moderate neuroprotective activity [14,15], neuroprotection during stroke remains a promising and relevant treatment strategy [15,16].

*c*-Jun N-terminal kinase (JNK) signaling is involved in the pathogenesis of ischemic stroke, along with multiple different signaling pathways [5]. The activation of JNK is observed after global and focal cerebral ischemia (FCI) in rats and mice [17,18,19,20,21,22,23,24], and various small-molecule JNK inhibitors have exhibited neuroprotective effects in animal models, including stroke [25,26,27,28,29]. Recently, we found that the specific JNK inhibitors IQ-1S and IQ-1L exhibited neuroprotective activity in mouse and rat models of FCI, and in a rat model of global cerebral ischemia [22,30,31,32]. 

Oxidative stress [33,34,35] and neuroinflammation [36] are involved in the pathogenesis of cerebral ischemia–reperfusion. Indeed, preclinical and clinical studies suggest that the neuroprotective activity of compounds in acute cerebral ischemia–reperfusion models can be enhanced if therapeutic compounds exhibit a combination of antioxidant and anti-inflammatory properties [35,37]. One such compound is a derivative of tryptanthrin (TRYP) (indolo[2,1-*b*]quinazolin-6,12-dione). TRYP is an alkaloid from higher plants and several marine organisms [38], with anti-inflammatory properties [39,40] and high blood–brain barrier (BBB) permeation potential [40]. Previously, we found that the TRYP derivative tryptanthrin-6-oxime (TRYP-Ox) was a very specific small-molecule JNK inhibitor and was more selective for JNK1/JNK3 vs. JNK2. In addition to the inhibition of JNK, we found that TRYP-Ox also had anti-inflammatory activity [39,41]. Thus, the goals of this research were to study TRYP-Ox as a neuroprotectant in a rat model of transient FCI and to confirm that TRYP-Ox inhibits JNK in vivo.

## 2. Results

### 2.1. BBB Permeability for TRYP-Ox (In Silico)

The calculated octanol–water partition coefficient (aLogP) for TRYP-Ox was 2.92, the topological polar surface area (tPSA) was 67.48 Å^2^, and the number of rotatable bonds (N_rot_) was 0. These values are in accordance with the model reported by Suenderhauf et al. [42] and indicate that TRYP-Ox can easily penetrate the BBB. Thus, TRYP-Ox is likely active in brain tissues and meets the Stroke Therapy Academic Industry Roundtable (STAIR) requirement for a neuroprotectant.

### 2.2. Effects of TRYP-Ox on Neurological Status

**Figure 1:**
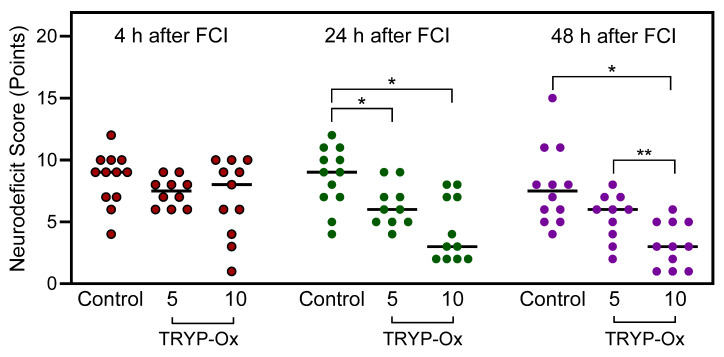
Analysis of neurological deficit in TRYP-Ox-treated rats. The mean scores of neurological deficits in FCI control rats (n = 12) and rats treated with 5 mg/kg TRYP-Ox (n = 10) and 10 mg/kg TRYP-Ox (n = 11) at 4 (red circles), 24 (green circles), and 48 h (purple circles) after FCI. * *p* < 0.05 for TRYP-Ox-treated vs. FCI control animals. ** *p* < 0.05 for rats treated with 10 vs. 5 mg/kg TRYP-Ox.

The surgical intervention did not change the neurological status of sham-operated animals (n = 5). The maximum neurological deficit score in the FCI control group was observed during the first day after surgical intervention and slightly decreased at 48 h after reperfusion (Figure 1). In contrast, the mean neurological deficit scores were progressively reduced in the TRYP-Ox-treated rats, indicating neuroprotection by this treatment. While the neurological status of rats administered TRYP-Ox was not significantly different from that of the FCI control group after 4 h, the neurological deficit scores were 49% and 35% lower than in the FCI control group for rats treated with 5 and 10 mg/kg TRYP-Ox, respectively, at 24 h after FCI. Likewise, the mean neurological deficit scores at 48 h after FCI were 41% and 28% lower than in the control group for rats treated with 5 and 10 mg/kg TRYP-Ox, respectively.

### 2.3. Effects of TRYP-Ox on Infarct Size 

**Figure 2:**
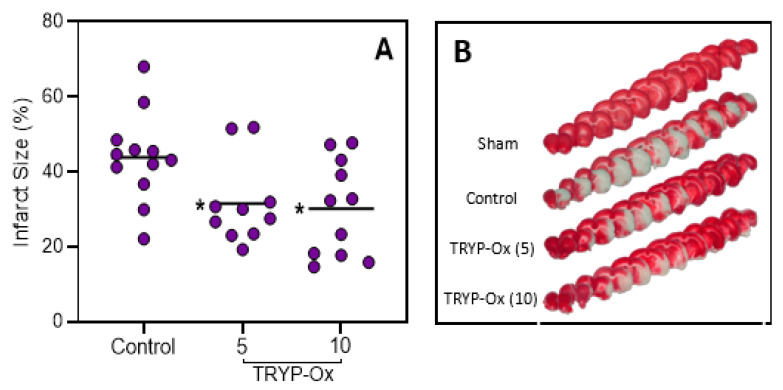
Effects of TRYP-Ox on infarct size. **Panel A**: Average mean swelling-adjusted sizes of cerebral infarction. Infarct size was measured in brains of FCI control rats (n = 12) and TRYP-Ox-treated rats at doses of 5 mg/kg (n = 10) and 10 mg/kg (n = 11) at 48 h after FCI. * *p* < 0.05 vs. FCI control animals. **Panel B**: Representative serial brain slices in rats of sham-operated, control, and experimental groups. White areas represent the regions of infarction in representative samples of 2,3,5-triphenyl tetrazolium chloride (TTC)-stained brain sections prepared from rats at 48 h after FCI.

The effects of TRYP-Ox on the magnitude of infarction in rats with FCI are presented in Figure 2. Macrofocal cerebral infarction developed in all animals of the FCI control group at 48 h after FCI. Brain swelling in the FCI control group was 18.5 ± 2.0%. In the group of rats administered 5 and 10 mg/kg of TRYP-Ox, brain swelling was 28% and 63% lower than that in the FCI control group, respectively. Importantly, the difference in the effects of TRYP-Ox on brain swelling between the experimental groups was significant (*p* < 0.05). The swelling-adjusted size of cerebral infarction in the control group was 43.8 ± 3.4% of the area of the affected left hemisphere. In the group of rats administered 5 and 10 mg/kg of TRYP-Ox, the swelling-adjusted size of cerebral infarction was 31.6 ± 3.6 and 30.2 ± 3.9, which was significantly lower than the corresponding parameter in the control group (Figure 2A). The infarct regions of representative samples of the stained brain sections are shown in Figure 2B. Significant positive correlations were obtained between neurological deficit and infarct size in the FCI control group (r = 0.93; *p* < 0.001) and in the groups of animals treated with 5 mg/kg (r = 0.83; *p* < 0.01) and 10 mg/kg of TRYP-Ox (r = 0.96; *p* < 0.001). 

### 2.4. Effects of TRYP-Ox on Brain Edema 

No interhemispheric differences in water content were observed in brain tissue from sham-operated rats (Table 1). At 24 h after FCI, water content in the left hemisphere, which was the site of infarction, increased in rats from the FCI control group by 30% vs. those values in sham-operated rats (*p* < 0.001) and reached 4.4 ± 0.1 g/g of dry weight. Water content in the contralateral right hemisphere was not changed significantly. The coefficient of interhemispheric asymmetry was 1.20 ± 0.03 (*p* < 0.001). Edema of the affected hemisphere occurred predominantly due to an increase in water content in supraventricular structures (by 40%, *p* < 0.01) and, to a lesser degree, in subventricular structures (by 19%, *p* < 0.01). In rats treated with 10 mg/kg of TRYP-Ox, water content in the infarction-affected hemisphere was decreased by 11% (*p* < 0.01) compared to the FCI control animals. However, the decrease in the coefficient of interhemispheric asymmetry did not reach the level of statistical significance. Edema of the affected hemisphere decreased mostly due to a decrease in water content in supraventricular structures (by 17%, *p* < 0.01) (Table 1).

### 2.5. Effect of TRYP-Ox on JNK Activity

**Figure 3:**
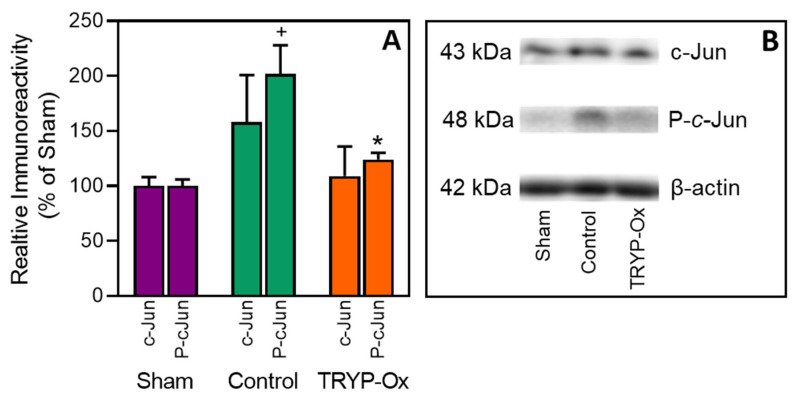
*c*-Jun phosphorylation in the cerebral cortex. Phosphorylation of *c*-Jun was evaluated in sham-operated rats (n = 3), FCI control rats (n = 3), and 10 mg/kg TRYP-Ox-treated rats (n = 3) at 1 h after FCI. **Panel A**: Semiquantitative results of the immunoreactivity for phospho-*c*-Jun (P-*c*-Jun) and total *c*-Jun, as determined by densitometric analysis of the immunoblots. The data are presented as a percentage relative to the group of sham-operated rats. ^+^ *p* < 0.05 vs. sham-operated; * *p* < 0.05 vs. FCI control rats. **Panel B**: Representative immunoblot bands from each group.

As shown in Figure 3, a two-fold increase in phospho-*c*-Jun (*p* = 0.049) expression was seen in the cerebral cortex in FCI control rats 1 h after FCI vs. that found in sham-operated animals. In contrast, the level of phospho-*c*-Jun in rats treated with 10 mg/kg TRYP-Ox decreased by 75% (*p* < 0.05) compared with the corresponding value in control rats. The values of total *c*-Jun expression did not significantly differ between the three groups of animals.

### 2.6. Effects of TRYP-Ox on the Levels of Cytokines in the Cortex

**Figure 4:**
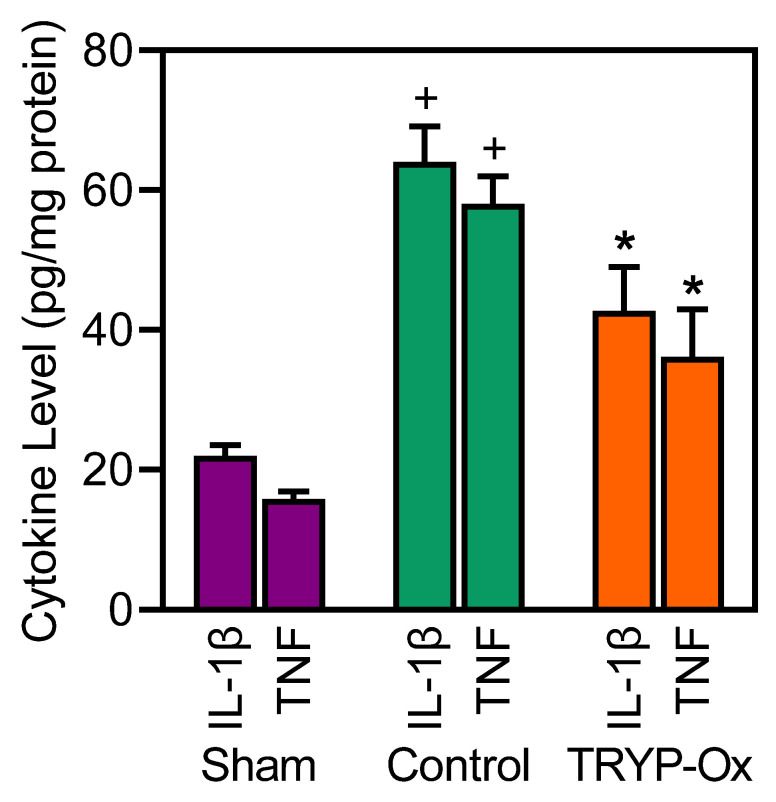
Effect of TRYP-Ox on cytokine levels in the cortex. Cytokine levels (pg/mg protein) were measured in the left cortex in sham-operated rats (n = 7), FCI control rats (n = 8), and 10 mg/kg TRYP-Ox-treated rats (n = 8) at 24 h after FCI. ^+^ *p* < 0.05 vs. sham-operated animals; * *p* < 0.05 vs. FCI control animals.

At 24 h after FCI, the levels of IL-1β and TNF in the cortex of ischemic focus increased by 2.9 and 3.7 times, respectively, in the rats of the FCI control group vs. those levels measured in the sham-operated animals (*p* < 0.05). During this period, the levels of IL-1β and TNF decreased by 33% and 38%, respectively, in rats treated with 10 mg/kg TRYP-Ox vs. those in the FCI control group (*p* < 0.05) (Figure 4).

### 2.7. Antioxidant Effect of TRYP-Ox 

The analysis of reactive oxidants in brain homogenates has long been used to measure the antioxidant activity of various compounds by chemiluminescence [43,44,45,46]. Chemiluminescence correlates with other lipid peroxidation assays, such as thiobarbituric acid reactant accumulation, and can be used to monitor the lipid peroxidation rates and the effects of treatments on lipid peroxidation [43,47,48].

**Figure 5:**
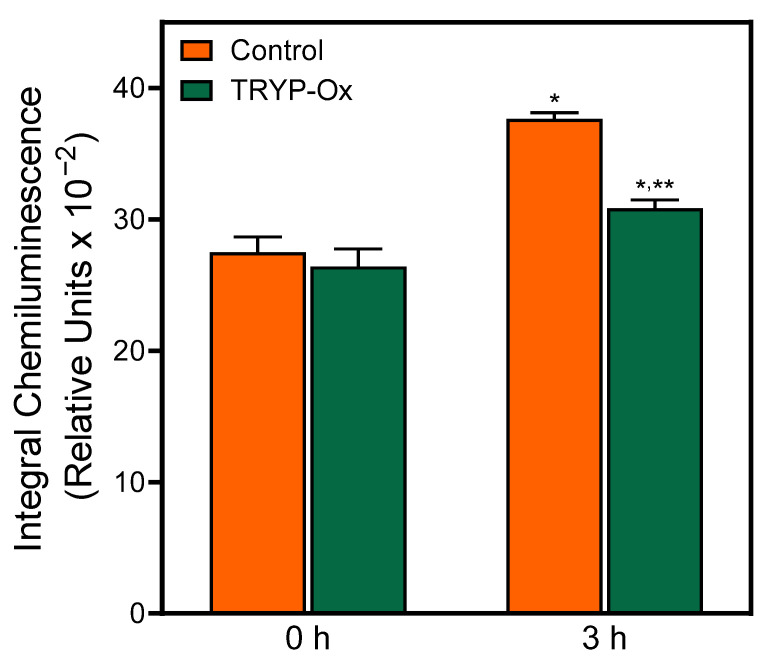
Effect of TRYP-Ox treatment on rat brain homogenate chemiluminescence. Brain homogenates were prepared from control rats treated with 20 µL of Tween 80 in 2 mL of isotonic NaCl solution (n = 5) and rats treated with 10 mg/kg TRYP-Ox (n = 5). Chemiluminescence was measured before (t = 0) and after incubation of the homogenates in the dark at 22 °C for 3 h. Integrated chemiluminescence (relative units) calculated over 60 s is shown. * *p* < 0.05 for t = 0 h vs. t = 3 h; ** *p* < 0.01 for TRYP-Ox-treated vs. FCI control at t = 3 h.

To evaluate the antioxidant effects of TRYP-Ox, chemiluminescence was analyzed in brain homogenates from rats treated with *i.p.* injections of vehicle (control group) and rats treated with *i.p.* injections of 10 mg/kg TRYP-Ox (Figure 5). Initial chemiluminescence in rat brain homogenates from both groups did not differ significantly (*p* = 0.60). After 3 h incubation, the level of chemiluminescence in the brain homogenates of control rats increased significantly by 37% (*p* < 0.01), whereas the brain homogenates of rats treated with TRYP-Ox exhibited a much smaller 17% increase in chemiluminescence, which was significantly lower than that in the control rats (*p* < 0.01).

## 3. Discussion

Successful phase II-IV clinical trials of novel neuroprotectants [37] and ongoing studies of other promising neuroprotectants [5] demonstrate that neuroprotection is still a relevant strategy in stroke [16,49]. Our work extends previous research focusing on the discovery and study of new candidate neuroprotectants for the treatment of ischemic stroke. In performing this research, we were guided by the STAIR XI Consolidated Recommendations 2021 [11] and considered the main requirements of stage A, namely candidate treatment qualification, including dose responses, histological and behavioral outcomes, target engagement, and BBB penetration.

BBB permeability is an important property of a substance required to exert neuroprotective effects [11,50]. A number of neuroprotectants have shown promise in treating ischemic stroke; however, the delivery of these agents into the brain is challenging [51]. Note that our calculations showed that TRYP-Ox penetrates the BBB well, supporting its potential as a candidate neuroprotectant.

Neuroprotective strategies for arterial recanalization after stroke (thrombolysis/thrombectomy) focus on the prevention of an increase in the ischemic core [52]. For the first time, we demonstrated that treatment with TRYP-Ox (5 and 10 mg/kg) decreased the infarction area and accelerated neurological recovery. Thus, the administration of TRYP-Ox showed pronounced neuroprotective effects. The dose–response relationship for TRYP-Ox was manifested in the effect of the compound on neurological deficits. A greater therapeutic effect of TRYP-Ox at a dose of 10 mg/kg was demonstrated by a more pronounced and prolonged (up to 48 h) reduction in neurological deficits in rats after FCI.

The relationship between the size of cerebral infarction and the degree of neurological deficit has not been completely resolved for the FCI model. Some studies have provided evidence for a positive correlation between neurological deficit and infarct size [53,54,55]. However, other authors point out that neurological deficit scores are not a reliable criterion for the prediction of infarct size [56] and that the correlation between neurological deficit and infarct volume is limited to the first 6 h following FCI onset [57]. The relationship between the infarct size and neurological deficit in FCI rats treated with neuroprotective agents is also inconclusive. For some compounds, the dose response is manifested by an effect on both the infarct size and neurological deficit [58]. However, in animals treated with p38 kinase inhibitor SB 239063, a dose–response effect was not observed [59]. In the present study, the dose–response effect was found for neurological deficits (Figure 1) but was absent in relation to the infarction size (Figure 2). We suggest that the reduction in neurological deficit is an integral process that depends on the reduction of the infarction as well as the penumbra zone. The observed relationship for doses of TRYP-Ox and neurological deficit may be associated with a more pronounced anti-edematous effect of the compounds at 10 mg/kg vs. 5 mg/kg. Indeed, treatment with 10 mg/kg TRYP-Ox reduced brain swelling more than 5 mg/kg TRYP-Ox. This idea is also supported by the known association of the neurological deficit’s index with cerebral edema in rats with FCI [60].

Neuronal cell death is involved in ischemic stroke pathogenesis [5,34]. The activation of mitogen-activated protein kinases is one of the pathological mechanisms leading brain cells to death in stroke [24]. JNKs play essential roles in a variety of neuropathological signaling events and are important in regulating the survival of brain tissues [28,61]. For example, JNK signaling pathways play essential roles in neuronal injury triggered by reperfusion-induced oxidative stress [24,28,62]. Multiple studies have shown that increased JNK phosphorylation and the upregulation of JNK-dependent signaling pathway have been observed in rodent brains after global and focal ischemia [18,19,20,63]. Three JNKs, which are designated as JNK1, JNK2, and JNK3, have been identified, and ~10 different splicing isoforms exist in mammalian cells [64]. Note, however, that JNK3 is almost exclusively found in the brain [65] and appears to play a critical role in *c*-Jun phosphorylation and apoptotic cell death [66]. Kinases of the JNK signaling pathway may be used as therapeutic targets in stroke [24], and non-peptide synthetic JNK inhibitors have been reported (e.g., AS601245, IQ-1S, SP600125, SR-3306, and SU3327) [28]. There are also a number of other protein and nonprotein molecules that have been reported to inhibit JNK interactions with substrates and/or cell organelles [18,28,61,67,68,69,70,71]. Furthermore, some JNK inhibitors have demonstrated neuroprotective activity in animal models of stroke [25,26,27,31,32,72].

The choice of TRYP-Ox for the study was justified by its reported JNK-inhibitory activity in vitro [41]. Indeed, TRYP-Ox is a highly specific non-peptide small-molecule JNK inhibitor, as it does not bind other kinases [41]. Furthermore, TRYP-Ox is selective for JNK1/JNK3 vs. JNK2. Thus, one of the objectives of our study was to confirm that TRYP-Ox inhibits JNK in vivo. Importantly, the JNK-inhibitory effects of TRYP-Ox in vivo should be confirmed during the period of JNK activity increase, which occurs in 0.5–1 h after ischemia–reperfusion [26,73]. While addressing this goal, we encountered a methodological problem. It is reasonably believed that efforts to save the brains of stroke patients should be aimed at protecting the penumbra zone, where neuroprotective therapeutic effects could occur [74]. However, we were not able to isolate the perifocal zone in FCI rats during the first few hours after recirculation because the infarction area could not be clearly visualized at the early stages of stroke injury. A clear distinction between the ischemic core and penumbra area occurs one day after FCI [75]. Therefore, the brain tissue for Western blot analysis was sampled from the middle cerebral artery (MCA) region.

In the present study, we demonstrated the inhibitory effect of TRYP-Ox on JNK in vivo and showed a decrease in phospho-*c*-Jun expression in the cerebral cortex early after brain reperfusion. These results confirmed the selective inhibitory effect of TRYP-Ox on JNK, which agrees with our earlier in vitro experiments [41]. Considering the role of JNK in cerebral ischemia–reperfusion injury [17,18,19,20,21,22] and data regarding the neuroprotective activity of other JNK inhibitors [22,24,28,31,32,66,69,71], we assume that JNK inhibition is one of the leading mechanisms of TRYP-Ox’s neuroprotective activity.

JNKs do not only phosphorylate *c*-Jun, and close to 100 JNK substrates are known [76]. However, *c*-Jun is a major JNK substrate, and *c*-Jun phosphorylation is closely tied to activator protein 1 (AP-1) activation, which is one of the common links to the activation of inflammatory genes and neuronal cell death [77]. Neuroinflammation is an important pathogenic factor in cerebral ischemia–reperfusion [36,78], and it has been reported that stroke activates the expression of pro-inflammatory cytokines, such as TNF and IL-1β [79]. Studies of the therapeutic potential of TRYP-Ox showed that it significantly attenuated inflammation in experimental models of arthritis [39]. Likewise, the JNK-binding activity of TRYP-Ox correlates with its ability to inhibit lipopolysaccharide-induced nuclear factor-κB/AP-1 activation in human monocytic THP-1-Blue cells and IL-6 production by human MonoMac-6 cells [41]. In the present study, we demonstrated for the first time in the FCI model the distinct anti-inflammatory properties of TRYP-Ox, as demonstrated by the decreased expression of pro-inflammatory cytokines IL-1β and TNF in the cortex of the ischemic focus. This may be due to the inhibitory effect of TRYP-Ox on the JNK3/*c*-Jun signaling cascade.

Antioxidant and anti-inflammatory effects are essential for the emergence of neuroprotective activity required in stroke treatment [34,35,80]. Therapeutic approaches using neuroprotectants with antioxidant properties are based on the concept of acute stroke pathogenesis, and some studies have confirmed the neuroprotective effects of antioxidants in cerebral ischemia [81,82]. Although the antioxidant activity is beneficial, it is insufficient for a molecule to be considered a neuroprotectant because most clinical trials of individual drugs with free radical scavenger activity have failed [12,83,84], although recent clinical studies have demonstrated the effectiveness of the antioxidant edaravone [85]. Our finding that TRYP-Ox limited the level of spontaneous chemiluminescence of brain tissue homogenates suggests the ability of this JNK inhibitor to also inhibit the free-radical oxidation of brain tissue. Inhibition of the chemiluminescence of the brain tissue homogenate not only demonstrated the presence of antioxidant properties in TRYP-Ox, but also confirmed its ability to penetrate through the BBB.

Free radicals play important roles in the pathogenesis of cerebral ischemia–reperfusion through their ability to target multiple cellular pathways [86,87], including the JNK signaling pathway [27,66,88,89]. In the context of our study, of particular interest are the data that some antioxidants (e.g., edaravone, N-acetylcysteine, and tetrahydroxystilbene glucoside) that protect nerve cells from cerebral ischemia–reperfusion injury can also regulate the JNK signaling pathway [21,86,90]. For example, antioxidant therapy with N-acetylcysteine attenuated JNK3 activation and subsequent neural damage in models of cerebral ischemia [91]. Therefore, the JNK3 signaling pathway is an important therapeutic target not only for specific JNK inhibitors but also for compounds with antioxidant activity. Based on this, it can be assumed that the antioxidant effects of TRYP-Ox are able to amplify the direct inhibitory effect of the compound on JNK during cerebral ischemia–reperfusion.

## 4. Materials and Methods

### 4.1. Animals

This study was performed in accordance with EU Directive 2010/63/EU regarding the protection of animals used for scientific research. The protocol of the study was approved by the Animal Care and Use Committee of the Goldberg Research Institute of Pharmacology and Regenerative Medicine, Tomsk NRMC (protocol 207012023, approved 30 January 2023). Experiments were performed using adult male Wistar rats (250–280 g) obtained from the Department of Experimental Biological Models of the Goldberg Research Institute of Pharmacology and Regenerative Medicine, Tomsk NRMC.

### 4.2. Chemicals, Drugs, and Kits

Propofol-Lipuro was from B. Braun Melsungen AG (Melsungen, Germany); Tween 80 was from Merck (Darmstadt, Germany); 10% neutral formalin was from BioVitrum (St. Petersburg, Russia); diethyl ether for anesthesia was from Kuzbassorgkhim (Kemerovo, Russia), ethanol was from Konstanta-Farm M (Moscow, Russia), ethyl acetate was from Component-Reactive (Moscow, Russia); 2,3,5-triphenyl tetrazolium chloride, dimethyl sulfoxide (DMSO), and rat TNF and rat IL-1β ELISA kits were from Sigma-Aldrich (St. Louis, MO, USA).

### 4.3. Compounds and Doses

TRYP was purchased from Combi-Blocks (San Diego, CA, USA), and TRYP-Ox was synthesized from TRYP, as described previously [92]. The structure of TRYP-Ox was confirmed by mass spectrometry and nuclear magnetic resonance (NMR); the sample purity was 99.9%. To prepare the TRYP-Ox suspension, the powder corresponding to a proper dose for the animal was aseptically pounded with 20 μL of Tween 80, and 2 mL of 0.9% NaCl solution was added.

Studies of promising neuroprotectants require the assessment of dose responses [11,93]. Two doses (5 and 10 mg/kg) of TRYP-Ox were selected to study the neuroprotective effects of this compound. The higher dose (10 mg/kg) was chosen based on the significant anti-inflammatory activity of TRYP-Ox at this dose when the compound was administered intraperitoneally (*i.p*.) in a mouse model of arthritis [39], and adjustment of the dose for rats was performed according to the guide for dose conversion between animals [94].

### 4.4. Molecular Modeling

According to the STAIR requirements for preclinical studies of promising neuroprotectants [11,93], the study must demonstrate that the drug reaches the target organ. Thus, direct neuroprotective effects require that a treatment can penetrate the BBB at concentrations sufficient to provide a therapeutic effect. Molecular modeling of TRYP-Ox was used to calculate the following parameters: octanol–water partition coefficient (aLogP), topological polar surface area (tPSA), and number of rotatable bonds (N_rot_). aLogP was calculated using the HyperChem 7 software based on an additive scheme [95]; tPSA was calculated according to atomic increments reported in [96]; and N_rot_ was determined from the structural formula. The ability of the compound to penetrate the BBB was assessed in accordance with the model proposed by Suenderhauf et al. [42]. 

### 4.5. Experimental In Vivo Protocols 

**Figure 6:**
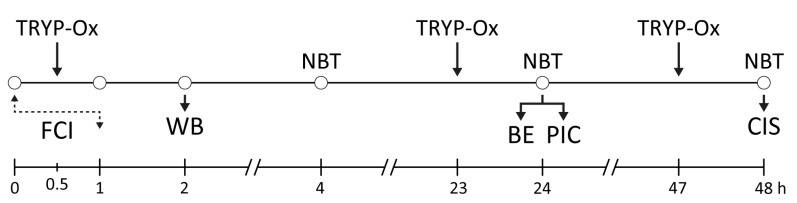
Schematic protocol of the in vivo study. TRYP-Ox, intraperitoneal injection of TRYP-Ox; FCI, focal cerebral ischemia; NBT, neurobehavioral tests; WB, Western blot analysis; BE, brain edema measurement; PIC, pro-inflammatory cytokine (IL-1β and TNF) analysis; CIS, cerebral infarct size assessment.

In vivo experiments were performed using 108 Wistar rats subjected to FCI modeling. Animals from sham-operated control and experimental groups were euthanized using a CO_2_ euthanasia device (Open Science, Krasnogorsk, Russia) 1, 24, and 48 h after modeling FCI. JNK inhibition in brain tissue was assessed 1 h after FCI. Brain edema and the levels of pro-inflammatory cytokines IL-1β and TNF were measured 24 h after FCI. Neurological status was assessed 4, 24, and 48 h following FCI onset. Cerebral infarction size was measured 48 h after reperfusion. Rats subjected to FCI were randomly divided into control and experimental(s) groups. JNK inhibition in brain tissue, brain edema, and the level of pro-inflammatory cytokines was measured in three groups: sham-operated, control, and experimental (10 mg/kg TRYP-Ox). Neurological characteristics and infarct dimensions were assessed in three groups: sham-operated, FCI control, and two experimental groups (5 and 10 mg/kg TRYP-Ox). FCI control rats received *i.p*. injections of 20 µL of Tween 80 in 2 mL of isotonic NaCl solution 30 min before reperfusion and then at 23 and 47 h after FCI. Animals in the experimental group were administered *i.p.* injections of 2 mL TRYP-Ox suspension at doses of 5 or 10 mg/kg, according to a similar schema. The schematic protocol of the study is presented in Figure 6.

### 4.6. Experimental Ex Vivo Protocol

Ex vivo experiments were performed using 10 Wistar rats. Rats in the control group (n = 5) received *i.p*. injections of 20 µL of Tween 80 in 2 mL of isotonic NaCl solution. Rats in the experimental group (n = 5) received *i.p*. injections of a 10 mg/kg TRYP-Ox suspension. Rats in each group were decapitated 3 h after *i.p*. injections under diethyl ether anesthesia. The brains were removed, and the cortex of the left hemisphere (100 mg) was dissected and homogenized in 1 mL of ice-cold 0.05 M KH_2_PO_4_∙NaOH buffer, pH 7.4. Homogenization was performed using a 20 G needle 10 times and then with a 22 G needle 5 times. Next, 950 μL of buffer was added to 50 μL of the homogenate, and integral chemiluminescence during 60 s was measured using a Lumat3 LB9508 luminometer (Berthold Technologies, Bad Wildbad, Germany) before and after 3 h incubation in the dark at 22 ± 1 °C [43].

### 4.7. Transient FCI Model

According to the STAIR recommendations [11,93], the efficacy of a new neuroprotectant should be assessed in an experimental model of cerebral ischemia based on histological and behavioral outcomes. In this study, intraluminal occlusion of the MCA in rats was used as an FCI model [97], and the animals were randomized before surgical procedures. General anesthesia was induced with propofol (10 mg/kg/h) administered through a catheter implanted in the right femoral vein while the animals were under brief ethyl ether anesthesia. Propofol is a recommended anesthetic for use in rat models of cerebral ischemia [98]. Transient FCI was produced by occluding the MCA for 1 h with a filament manufactured by the Doccol Corporation (Sharon, MA, USA). The filament was then removed, a ligature was placed in the external carotid artery, and blood flow was restored through the left internal carotid artery. Success of the occlusion was confirmed by the severity of the neurological deficit and volume of the infarct. Animals in the sham group underwent a similar surgical procedure but without insertion of the filament into the MCA. The body temperature of the animals during surgical procedures was maintained at 37.0 ± 0.05 °C using a HB 101/2 temperature control unit (Panlab Harvard Apparatus, Barcelona, Spain) and a homeothermic blanket control unit (Harvard Apparatus, Holliston, MA, USA). After occlusion of the MCA, the animals were again randomized into three groups: an FCI control group and two experimental groups (TRYP-Ox at doses of 5 and 10 mg/kg). The surgical wound was closed, and the animals that recovered from anesthesia were transferred to their cages and received free access to chow and water. Postsurgical mortality of rats was 10% (n = 12 dead rats from the 120 total rats). Animals that did not survive after surgery were excluded from the studies. Postsurgical animals in severe condition and experiencing pain incompatible with further experiments were euthanized (n = 5 animals from the 108 total number of postsurgical rats). Thus, the combined number of rats excluded from studies by mortality or severe condition was 17 animals. All rats had neurological deficits and infarct volumes after MCAO surgery.

### 4.8. Neurological Deficit Evaluation

Rat neurological status was assessed by a researcher who was unaware of the group assignment. Neurobehavioral tests were performed 4, 24, and 48 h after reperfusion. The degree of neurological abnormalities was determined by a modified neurologic severity score based on motor tests, sensory tests, beam balance tests, and reflexes absent or abnormal movements [99]. Chen et al. [99] described in detail the test procedures that we strictly followed while assessing neurological deficits. The severity of neurological deficit was rated by the total score obtained in all tests (0–18 points).

### 4.9. Cerebral Infarct Size Assessment

Cerebral infarct size was examined by the experimenter using the “blind method”. To assess infarct size following FCI, the rat brains were frozen at −12 °C for 2.5 h and cut into 1.3-mm-thick frontal slices using an array of 17 histological knives assembled as a unit. The brain slices were left to thaw at room temperature, followed by incubation for 15 min in a 0.5% solution of TTC at 37 °C in the dark. The cerebral slices were fixed for 15 min in 10% buffered formalin, mounted on glass slides, and then scanned to obtain images at 600 dpi resolution using a HP Scanjet 3770 with HP Director software version 43.1.6.000. Images were stored in *.tiff format and processed using the Adobe Photoshop 6.0 software. On each slide, the infarction zone and the zone of unaffected tissue were measured in pixels, and then these zones were summed. The difference in the area of the affected and contralateral hemispheres was regarded as % swelling of the brain tissue [100]. The area of cerebral infarction was expressed as a percentage of the total area of the infarcted hemisphere corrected for brain swelling using the following formula: the infarct size in the infarcted hemisphere × (the contralateral hemisphere size/the infarcted hemisphere size) [101].

### 4.10. Brain Edema Measurement

Rats of each group were decapitated 24 h after FCI under diethyl ether anesthesia. The brains were removed and divided into 3 parts: cerebellum, left hemisphere, and right hemisphere. Each hemisphere was also divided according to the level of the brain ventricles into supraventricular and subventricular parts. The brain samples including the cerebellum were weighed to obtain the wet weight and were then dried at 105 °C to a constant weight. The cerebellum was detached to serve as a control [102]. Brain water content (g/g dry weight) was calculated by the wet and dry method as follows: (wet weight − dry weight)/dry weight [103]. To assess interhemispheric differences in H_2_O content, the coefficient of interhemispheric asymmetry was calculated as left (ischemic) hemisphere water content/right (contralateral) hemisphere water content.

### 4.11. Analysis of IL-1β and TNF Levels in Brain

For brain tissue preparation, rats were decapitated under deep anesthesia with diethyl ether at 24 h after reperfusion. The ischemic core area of the cortex of the left MCA region was dissected, frozen, and kept at −80 °C until use. At the time of analysis, the samples were defrosted and homogenized in ice-cold cell lysis buffer. The homogenates were centrifuged at 10,000 rpm for 10 min at 4 °C. The supernatants were collected and used for the analysis of IL-1β and TNF levels, according to the manufacturer’s instructions. All data are expressed as pg of cytokine per mg of protein. The determination was performed using a ChemWell 2910 biochemistry analyzer.

### 4.12. Western Blot Analysis

Based on previously reported studies, we selected time points for Western blot analysis [18,20,25]. FCI control and experimental rats were euthanized 1 h after reperfusion, and sham-operated rats were euthanized 2 h after surgery. Cerebral cortex tissue from the MCA pool was isolated from the left hemisphere on an ice plate, homogenized, and quickly frozen in liquid nitrogen. Twenty mg of tissue homogenate was ground with 200 μL of ice-cold RIPA lysis buffer (Abcam, Cambridge, UK) containing protease and phosphatase inhibitor cocktails (Sigma-Aldrich) and sonicated. The homogenates were then centrifuged at 15,520 g and 4 °C for 20 min, and the supernatants were collected to determine the total protein concentration using a Quick Start Bradford Protein Assay (Bio-Rad, Richmond, CA, USA). The samples (40 μg) were diluted in sodium dodecyl sulfate (SDS)-loading buffer, boiled at 95 °C for 5 min, separated by 10% SDS-polyacrylamide gel electrophoresis, and transferred to polyvinylidene difluoride membranes (Immune-Blot, Bio-Rad) using a semi-dry electrophoretic transfer system (Trans-Blot SD, Bio-Rad). The membranes were blocked in 5% bovine serum albumin (BSA) (Sigma-Aldrich) in Tris-buffered saline (20 mM Tris and 150 mM NaCl) containing 0.1% Tween 20 at room temperature for 1 h and incubated with rabbit monoclonal anti-*c*-Jun (60A8) and anti-phospho-*c*-Jun (Ser63) (54B3) primary antibodies (both from Cell Signaling, Danvers, MA, USA) diluted in the blocking solution (1:1000) overnight at 4 °C. The membranes were then incubated with secondary horseradish peroxidase-coupled goat anti-rabbit IgG antibodies (Cedarlane, Burlington, ON, Canada) 1:10,000 in Tris-buffered saline containing 0.1% Tween 20 and 1% BSA for 1 h. Anti-β-actin-peroxidase monoclonal antibodies (Sigma-Aldrich) were used as a loading control. Proteins were detected by chemiluminescent peroxidase substrate Immobilon Western (MilliporeSigma, Burlington, MA, USA) and the optical density of each band was determined by G:BOX ChemiXRQ and Gene-Tools (4.3.9.0) software (Syngene, Cambridge, UK). Values were normalized by β-actin and expressed as the relative optical density.

### 4.13. Statistical Analysis

Statistical analysis was performed with the Statistica 8.0 software and GraphPad Prism 9.0 (GraphPad Software, San Diego, CA, USA). All results are expressed as mean ± SEM. The data were tested for normal distribution with Shapiro–Wilk’s W test. Group variation was assessed with the Kruskal–Wallis test. Significant differences between the variables were assessed with the Mann–Whitney U-test. Values were considered statistically significant when *p* was <0.05. Linear regression analysis was performed to determine the relationship between the infarct size and neurological deficit. The quality of the fit was assessed by the Pearson correlation coefficient (r).

## 5. Conclusions

In summary, we confirmed that TRYP-Ox exerted a pronounced neuroprotective effect in the rat FCI model. Neuroprotective effects of TRYP-Ox occurred at doses of 5 and 10 mg/kg. The mechanisms of TRYP-Ox’s neuroprotective effects included JNK inhibition and antioxidant and anti-inflammatory activity. Thus, our data suggest that TRYP-Ox may be considered as a promising compound for the development of an effective neuroprotectant to treat stroke. These results substantiate the expediency of further in-depth studies of TRYP-Ox, including the immunohistochemical analysis of glial activation (microglia and astrocytes), changes in various types of cells in the damaged areas, and the level of cell death, as well as additional studies of the mechanisms of antioxidant activity in neuronal cell models.

## Figures and Tables

**Table 1:** Effects of TRYP-Ox on the brain water content in brain hemispheres.

Brain Hemisphere		Sham-Operated	FCI Control	TRYP-Ox
Left Hemisphere	Total	3.4 ± 0.1	4.4 ± 0.1 *	3.9 ± 0.1 *+
	Supraventricular part	3.5 ± 0.1	4.9 ± 0.1 *	4.1 ± 0.1 *+
	Subventricular part	3.1 ± 0.1	3.7 ± 0.1 *	3.6 ± 0.1 *
Right Hemisphere	Total	3.4 ± 0.1	3.7 ± 0.1	3.5 ± 0.1
	Supraventricular part	3.5 ± 0.1	3.7 ± 0.1	3.7 ± 0.1
	Subventricular part	3.1 ± 0.1	3.4 ± 0.1 *	3.4 ± 0.1 *
Cerebellum		3.4 ± 0.1	3.4 ± 0.04	3.4 ± 0.02

Brain water content (g/g dry weight) was measured in sham-operated (n = 9), control (n = 9), and 10 mg/kg TRYP-Ox-treated (n = 8) rats at 24 h after FCI. * *p* < 0.05 vs. sham-operated animals; ^+^ *p* < 0.05 vs. FCI control animals.

## Data Availability

Data is contained within the article.

## Abbreviations

BBB, blood–brain barrier; FCI, focal cerebral ischemia; IL, interleukin; JNK, c-Jun N-terminal kinase; MCA, middle cerebral artery; TNF, tumor necrosis factor; TRYP, tryptanthrin; TRYP-Ox, tryptanthrin-6-oxime; TTC, 2,3,5-triphenyl tetrazolium chloride.
